# Understanding the Role of Shape and Composition of Star-Shaped Polymers and their Ability to Both Bind and Prevent Bacteria Attachment on Oral Relevant Surfaces

**DOI:** 10.3390/jfb10040056

**Published:** 2019-12-17

**Authors:** Hamid Mortazavian, Guillaume A. Picquet, Jānis Lejnieks, Lynette A. Zaidel, Carl P. Myers, Kenichi Kuroda

**Affiliations:** 1 Department of Biologic and Materials Sciences & Prosthodontics, School of Dentistry, University of Michigan, Ann Arbor, MI 48109, USA; mortazavian.hamid@gmail.com (H.M.); jaanislejnieks@gmail.com (J.L.); 2 Oral Care Early Research, Colgate-Palmolive Company, Piscataway, NJ 08855, USA; guillaume_picquet@colpal.com (G.A.P.); Lynette_Zaidel@colpal.com (L.A.Z.)

**Keywords:** polymer, dental, antibacterial, antifouling, hydroxyapatite, star-shaped, hydrophobicity, acrylic acid, oral, composition

## Abstract

In this study, we have prepared a series of 4- and 6-arm star-shaped polymers with varying molecular weight and hydrophobicity in order to provide insight into the role and relationship that shape and composition have on the binding and protecting of oral relevant surfaces (hydroxyapatite, HAP) from bacteria colonization. Star-shaped acrylic acid polymers were prepared by free-radical polymerization in the presence of chain transfer agents with thiol groups, and their binding to the HAP surfaces and subsequent bacteria repulsion was measured. We observed that binding was dependent on both polymer shape and hydrophobicity (star vs. linear), but their relative efficacy to reduce oral bacteria attachment from surfaces was dependent on their hydrophobicity only. We further measured the macroscopic effects of these materials to modify the mucin-coated HAP surfaces through contact angle experiments; the degree of angle change was dependent on the relative hydrophobicity of the materials suggesting future in vivo efficacy. The results from this study highlight that star-shaped polymers represent a new material platform for the development of dental applications to control bacterial adhesion which can lead to tooth decay, with various compositional and structural aspects of materials being vital to effectively design oral care products.

## 1. Introduction

The control and reduction of oral biofilm formation [1], initiated by bacterial species living in polymicrobial, pathogenic colonies at or below the gingival margin [2], are critical steps toward the prevention of dental caries and periodontal diseases [3,4,5]. While many methods have been proposed to prevent or treat these biofilms [6,7,8,9,10], one promising strategy is the use of synthetic polymer additives that bind to the tooth surface to act as a barrier or deterrent to the deposition of planktonic bacteria through either lethal [11,12,13] or non-lethal [14,15] mechanisms. Dental materials, especially those delivered from common over the counter products such as toothpaste or mouthwash, must effectively be multifunctional materials in that they must (1) deposit and stick to a tooth surface, (2) act as a barrier against bacteria attachment, (3) be robust against external challenges such as food and drink in order to not require constant reapplication, and (4) perform steps 1–3 in the presence of the salivary pellicle. Conventional polymers used for this strategy include poly (methyl vinyl ether/maleic acid) (Gantrez) and cross-linked micron-sized polyacrylic acid particles (carbopol). Additional polymers such as polyaspartate adhered to hydroxyapatite (HAP) and reduced the attachment of *Streptococcus sanguinis* [16]. While these materials do show the ability to reduce bacterial attachment to the tooth surface, the relationship between a polymer’s structure and composition to maximize efficacy, i.e., to bind to an enamel surface and provide anti-attachment properties, remains disconnected.

We have previously shown that star-shaped polymers with pre-assembled poly(hydroxyethyl methacrylate) (HEMA) chains formed stable polymer coatings on polyethylene terephthalate surfaces [17]. Consistent with similar reports [18,19,20,21,22,23,24,25,26,27,28,29,30,31,32], these star-shaped architectures provided brush-like structures of highly packed polymer chains that could physically repel bacteria to result in a significant reduction of attached bacteria. While this approach using water-insoluble polymers was suitable for hydrophobic resin surfaces, those potentially delivered from common oral care products are required to have significant water solubility. Materials, such as poly(acrylic acid) (PAA) are indeed water soluble, and have been known to bind to the tooth or HAP surfaces through interactions between the anionic carboxylate (COO-) groups in the polymer side chains and cationic calcium ions at the enamel surface [33,34,35].

**Figure 1:**
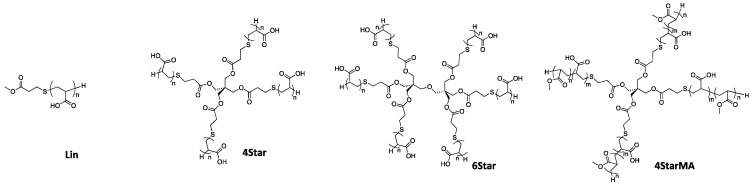
Star-shaped and linear poly(acrylic acid)s used in this study. The chemical structures of linear poly(acrylic acid) polymers (Lin), 4-arm (4Star) and 6-arm (6Star) star-shaped poly(acrylic acid), and 4-arm star-shaped copolymers with methyl acrylate (4StarMA).

As illustrated in Figure 1, we have prepared a small library of linear and star-shaped water-soluble polymers comprising of acrylic acid (AA) and methyl acrylate (MA) in order to draw a relationship between polymer structure (linear vs. 4-arm vs. 6-arm) and polymer composition (variable hydrophobicity) with their ability to bind to oral-relevant surfaces and provide bacterial anti-attachment in model systems. The specific goal of this report is to increase fundamental understanding of polymer-tooth surface interactions toward the development of new polymer platforms and products for anti-bacterial attachment activity.

## 2. Results and Discussion

### 2.1. Synthesis of 4- and 6-Arm Star-Shaped Polymers and Characterization

**Figure 2:**
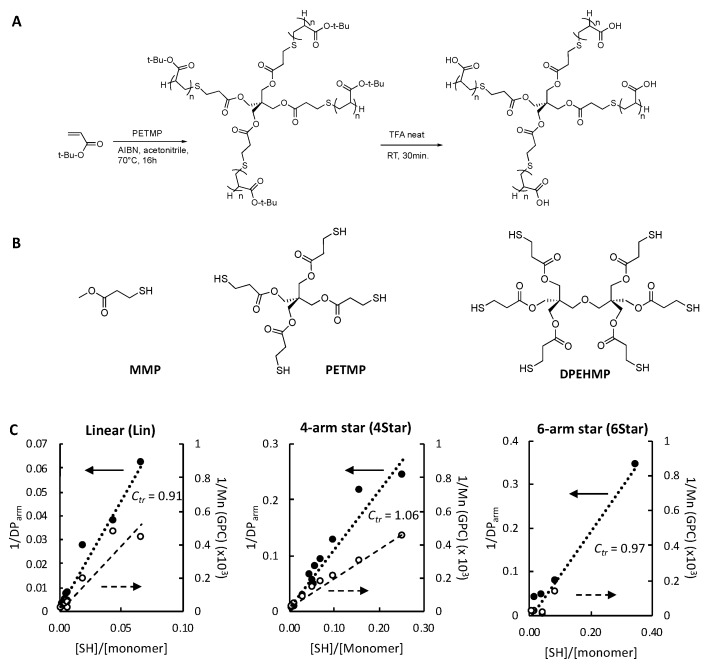
Synthesis of star-shaped polymers. (**A**) Synthesis of 4-arm star-shaped polymers, (**B**) Chemical structures of chain transfer agents. MMP: methyl 3-mercaptopropionate, PETMP: pentaerythritol tetrakis(3-mercaptopropionate), DPEHMP: dipentaerythritol hexakis(3-mercaptopropionate). (**C**) Mayo plots. The broken lines present the results of line fitting. *C_tr_* was determined from the slope of the line. [SH]/[monomer] = (The number of thiol groups in a CTA) *×* [CTA]/[monomer].

We wanted to test the free-radical polymerization in the presence of chain transfer agents (CTAs) as a facile synthetic strategy to prepare star-shaped polymers (Figure 2A) (See Tables S1–S6 in Supplementary Materials for polymerization conditions). In this polymerization, the thiol of a CTA reacts (R-SH) with the radical at the polymer chain and terminates the chain propagation by transferring the hydrogen atom (Figure S1). At the same time, the thiyl radical (R-S•) is generated and initiates new polymerization with the remaining monomers. This chain transfer cycle continues to consume all the remaining monomers, and the molecular weight of polymers can be determined by the relative reactivity of the radicals to CTAs compared to the monomers (chain transfer coefficient, *C_tr_* in Equation (2)) (See Section 3.5. Analysis of Polymerization Process in Materials and Methods for Equation (2)) and the molar ratio of CTA to monomers ([SH]/[monomer]). We have previously prepared star-shape polymers with 10–12 polymer chain arms by crosslinking the end groups of pre-existing polyHEMA polymer chains [17]. However, this preparation method required multiple synthesis and purification steps. To that end, we synthesized a series of polymers with a range of molecular weights by altering the ratio of CTA to monomers to determine if this synthetic method can provide star-shaped polymers with sufficient size control. In general, star-shaped polymers have been synthesized by living radical polymerization methods, which provided well-defined polymers [36]. However, we chose free-radical polymerization with thiol CTAs because we are interested in a facile approach, potentially capable of large-scale production.

The CTAs are small compounds with 4 or 6 thiol groups (PETMP and DPEHMP) (Figure 2B), which serve as core molecules to initiate propagation of polymer chains and yield star-shaped polymer structures. The mono-thiol chain transfer agent MMP provided linear polymers. We used *tert*-butyl acrylate (tBuA) as a protecting group of acrylic acid to facilitate polymer synthesis, and characterization through GPC and NMR spectroscopy. The average degree of polymerization of each arm (DP_arm_) was determined by comparing the integrated peak area from the polymer backbone to that of CTAs in the ^1^H NMR spectrum (See Experimental for details). As the ratio of CTA to monomers was increased, the molecular weight of polymers decreased, giving a series of star-shaped and linear polymers with *M_n_* of ~2000 to 200,000 g/mole (See Table S7 for the molecular weight of polymers). It should be noted that the molecular weights of polymers were further measured by size exclusion chromatography (SEC), which separates materials of various masses by the hydrodynamic volume of polymer chains. In general, the hydrodynamic volume of star-shaped polymer is smaller than that of linear polymer with same absolute molecular weight [37]. Therefore, the comparison of molecular weights between the linear and star-shaped polymers by SEC alone would not be sufficient.

To probe the chain transfer polymerization, we examined the relationship between the ratio of the thiol groups to monomers and the polymer chain length. The Mayo plots (1/DP or 1/Mn (GPC) vs. [SH]/[monomer] based on Equation (2) for linear, 4-arm, and 6-arm star-shaped polymers showed linear correlations (Figure 2C), and the *C_tr_* value of each CTA was determined as the slope of fitted lines in the Mayo plots (*C_tr_* = 0.91 (Lin), 1.06 (4Star), 0.97, (6Star)). In addition, the plots of 1/*M_n_* (determined by GPC) against [SH]/[Monomer] also showed linear correlations (Figure 2C). These results suggested that the polymerization was driven by independent chain transfer processes initiated by each thiol group of the CTAs. This further suggested that a polymer chain grew from each CTA arm (formation of star-shaped polymers), and the average polymer chain arm length could be controlled by varying the ratio of CTA to monomers.

The protected t-BuA polymers were treated with TFA to yield acrylic acid polymers (Figure 1 and Figure 2). Because the resultant acrylic acid polymers were no longer soluble in GPC solvent (THF), the molecular weights and distribution of polymers were not determined. The DP of resultant acrylic acid polymers could also not be relatively determined by ^1^H NMR analysis because the signals from the CTA agents were very small or not detected, which is likely due to low solubility of polymers in solvent. The linear, 4-arm, and 6 arm star-shaped polymers are denoted as Lin-X, 4Star-X, and 6Star-X, respectively, where X indicates the DP of each arm determined for the protected t-BuA polymers. 

We also extended the synthetic approach to the preparation of hydrophobic random copolymers. tert-Bu acrylate (tBuA) was co-polymerized with methyl acrylate (MA) to give random copolymers with acidic carboxylic and methyl (ester) groups in the side chains (4StarMA) (Table S8). The mole percentages of MA in the polymers were close to the initial feed ratios, indicating that the MA monomers were quantitatively incorporated to the polymer chains. The copolymers are denoted as 4StarMAY-X, where X and Y indicates the DP of each arm and mole percentage of MA in a polymer, respectively.

### 2.2. Binding of Star-Shaped Polymers to HAP

We first investigated the binding behaviors of star-shaped polymers onto HAP as a tooth surface model as it has a similar chemical composition to enamel [38,39]. The intrinsic binding properties of polymers can provide useful insights into the relationship between polymer structure and surface activity. Specifically, the binding constant of the polymers and the maximum amount of binding sites on hydroxyapatite surfaces would represent the polymers binding properties. Such information would be helpful to predict polymer activity and design new polymers for subsequent improvements.

**Figure 3:**
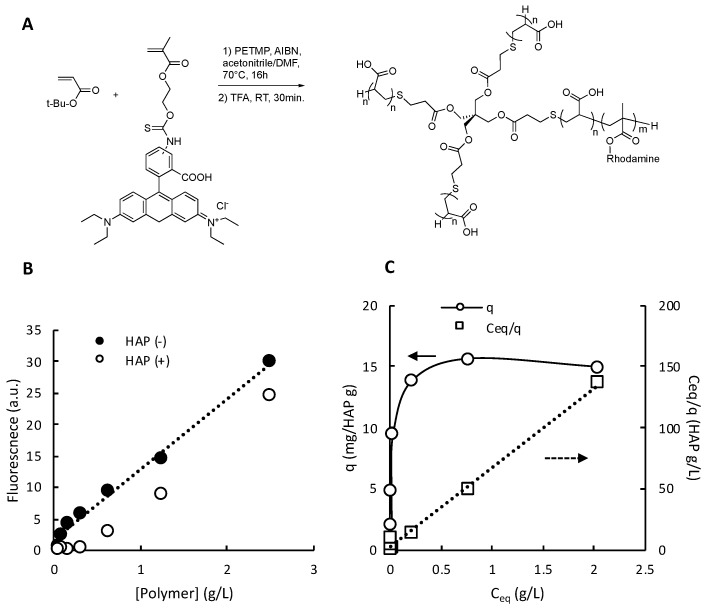
Synthesis of fluorescent dye-labeled polymers and their adsorption on hydroxyapatite powder (HAP). (**A**) Synthesis of rhodamine-labeled 4-arm star-shaped polymers. The rhodamine monomer (0.1 mol.% to the total number of monomers) was polymerized with t-Bu methacrylate. (**B**) Adsorption of 4-arm star-shaped polymer F-4Star-192 on HAP surfaces. Fluorescence intensities from supernatants of polymer assay solutions with and without HAP. (**C**) Adsorption isotherm and linear Langmuir plot. q: the amounts of polymers adsorbed onto the HAP surface. *C_eq_*: the polymer concentration of supernatant at equilibrium.

We synthesized rhodamine-labeled polymers (Figure 3A and Table S9) for the fluorescence-based binding assay described below. The assay used HAP powder dispersed in an aqueous solution as a model for HAP surfaces for polymer binding [40,41]. This assay provided a facile high-throughput method to determine the amounts of polymers that remained free in supernatant at equilibrium (*C_eq_*) and adsorbed onto the HAP surface (*q*) (Figure 3B). The amount of polymers adsorbed onto the HAP surface was increased as the polymer concentration was increased and appears to level off at high concentrations (Figure 3B). The adsorption isotherms may be represented by the following equation for the Langmuir adoption model:

(1) $$\frac{C_{eq}}{q}=\frac{C_{eq}}{q_{max}}+\frac{K_{d}}{q_{max}}$$

where *q_max_* and *K_d_* are the maximum amount of adsorbed polymers and dissociation constant, respectively [42,43,44]. The data were well fitted by Equation (1) (Figure 3C). The *q_max_* and *K_d_* values were calculated from the slope and intercept of each plot (Table S10). To compare the molecular behaviors of the star-shaped and linear polymers with different molecular sizes, we use the *q_max_* and *K_d_* values given in molar concentrations (µmol/g HAP and µM) for discussion, which present the binding behaviors of each polymer molecule. It should be noted that we used the DP of protected tBu polymers to calculate the *M_n_* values of de-protected polymers because of the difficulty to determine the DP of de-protected polymers by ^1^H NMR as described above. The *M_n_* values were used to convert the *q_max_* and *K_d_* values given in weight-based concentrations to molar concentrations.

**Figure 4:**
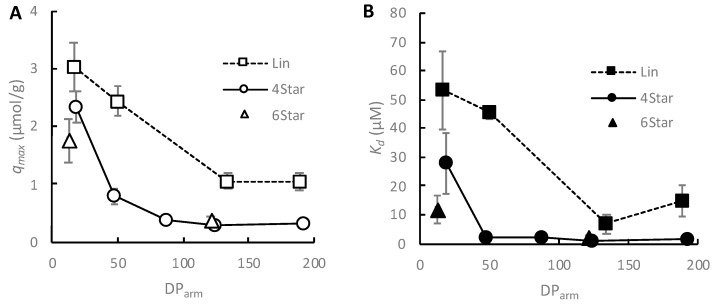
HAP adsorption of linear and 4-ram star-shaped polymers. (**A**) *q_max_* and (**B**) *K_d_* were determined by the Langmuir plot.

First, we examined the binding properties of star-shaped and linear polymers which have a range of DPs of each polymer arm in order to evaluate the effects of assembly of polymer chains on their HAP binding as well as the effect of polymer arm length (DP) on their binding behavior. In general, the *q_max_* values for 4- and 6-arm star-shaped polymers were smaller than that of the linear polymer (Figure 4A). This is likely because of the larger molecular sizes of star-shaped polymers, which occupy larger areas on the hydroxyapatite surface than the linear polymer, such that fewer star-shaped polymers could be bound to the hydroxyapatite surface. On the other hand, the dissociation constant *K_d_* values of star-shaped polymers were smaller than that of the linear polymer (Figure 4B), indicating that the star-shaped polymers adsorbed on the hydroxyapatite surface more strongly than the linear polymer. This can be explained by the large polymer sizes of star-shaped polymers which have more contact points on the hydroxyapatite surface for binding. The 4- and 6-arm star-shaped polymers with DP~120 showed the similar *q_max_* and *K_d_*, indicating that these polymers occupy similar areas on the HAP surfaces and have similar binding. 

Regarding the effect of polymer arm length (DP) on their binding behavior, the *q_max_* and *K_d_* value of 4-arm star-shaped polymers decreased as the DP of arms increased and leveled off at large DPs (Figure 4A). On the other hand, the *K_d_* value also leveled off for the polymers with large DPs (Figure 4B). The *q_max_* and *K_d_* values of linear polymers also decreased and appeared to level off at large DPs. These results suggest that the maximum number of adhered polymers and their binding affinity did not increase once the size of polymers became sufficiently large. This leveling-off of HAP binding behavior of the polymers may be explained by the following model. The anionic carboxylic groups of the polymer side chains are the binding ligand to HAP surfaces through electrostatic interactions. Therefore, as the polymer chains become longer, having more carboxylic side chains, the binding affinity of polymers for HAP would increase. However, the binding of carboxylic side chains to HAP surfaces requires the polymer chains to be flattened and/or stretched on the HAP surface, which is not favorable because of the large entropic penalty. Therefore, the binding of polymers would be determined by the balance between the two driving forces to maximize the number of binding sites by carboxylic groups on the HAP surface (enthalpy gain) and minimize the strain on polymer chains (entropic penalty). As the DP of the polymers increase, the number of carboxylic side chain groups increase, thus increasing their binding. However, once the polymers are long enough, the polymer chains would be difficult to be constrained on the HAP surface because of the entropic penalty, resulting in the leveling of *q_max_* and *K_d_*.

**Figure 5:**
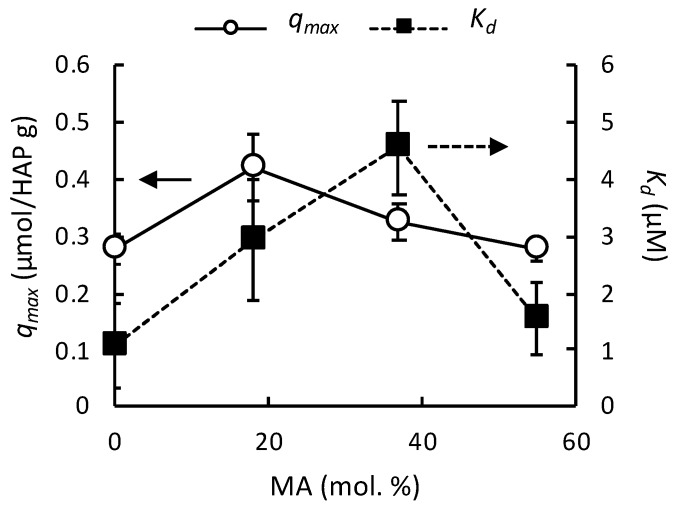
Effect of hydrophobic monomer composition on HAP adsorption of 4-arm star-shaped polymers.

The effect of hydrophobic side chains on polymers binding to HAP surfaces was also examined. The random copolymers with hydrophobic monomer MA showed maximum points in the *q_max_* and *K_d_* values as the composition of MA was increased (Figure 5). This binding behavior with maximum points may be explained by the interplay between the electrostatic binding of carboxylate groups to HAP and the intramolecular and intermolecular associations of MA groups. Increasing the MA composition reduces the number of carboxylic side chains, which may in turn reduce the binding affinity of polymers (higher *K_d_*). On the other hand, the hydrophobic groups may associate intramolecularly (within the same star-shaped polymer), which may prevent the extension of polymer chains for binding, resulting in low binding affinity (higher *K_d_*). Based on this model, the increase in the *K_d_* values for the low percentage of MA may indicate that the intramolecular association and/or reduced number of acidic groups are dominant, but low *K_d_* value for the polymer with 55% MA indicates the intermolecular hydrophobic association between star-shaped polymers may play an important role to stabilize the polymer layer. On the other hand, the *q_max_* also slightly increased, indicating the conformation of bound polymer chains are more compact (smaller occupied surface area). The polymers with 55% MA showed lower *q_max_*, indicating the polymer chains are more expanded likely because of increased intermolecular associations of MA groups between the star-shaped polymers, which is in good agreement with the low *K_d_* value. These results suggest that the binding behaviors of polymers to HAP surfaces can be controlled by their hydrophobicity, but is a less contributing factor than the overall shape (linear vs. star) of the polymer.

### 2.3. Anti-Bacterial Attachment Activity of Linear and Star-Shaped Polymers

**Figure 6:**
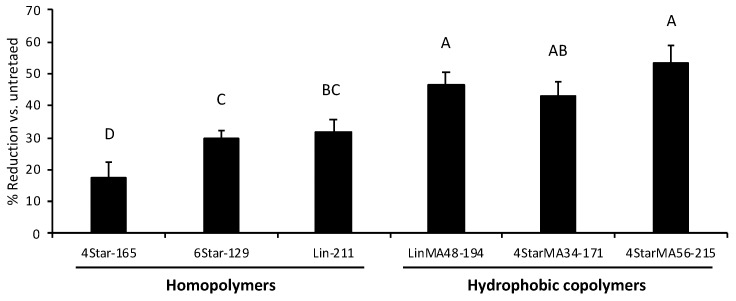
Anti-bacterial attachment activity of linear and star-shaped polymers. The activity of polymers was assessed by the percent reduction in bacterial attachment relative to control (untreated HAP surface). The data and error bars represent the average of 12 replicates with 95% confidence limits. The alphabetical letters on the bars present statistical grouping.

We have shown that shape significantly impacts the binding constant of polymers to HAP surfaces and that higher DPs are required for linear polymers than star-shaped in order to reach minimum *K_d_* values. As such, we selected a subset of linear, star-shaped, and hydrophobic polymers based on their respective DP values that were similar to their tightly-binding fluorescent counterparts (DP = 100–200) in order to choose those materials with the strongest affinity to the HAP surface (Figure 6 and Table 1). In this way, we have effectively normalized to the polymers’ *K_d_* such that any differences in anti-attachment or contact angle measurements could be ascribed to the shape and composition of the polymers rather than simply their lack of presence through dissociation from the HAP surface. Table 1 lists the non-fluorescent polymers chosen for further evaluation. Additionally, we have intentionally chosen materials that do not kill bacteria but instead repel. Surface modifications that reduce bacteria deposition and colonization through contact kill or simple cell repulsion will ultimately appear the same, i.e., both surfaces will have a sufficiently reduced amount of living bacteria. We acknowledge that a combination of mechanisms, that is, kill + repel, would likely have the greatest efficacy, however by using materials known to not kill, but rather repel, we can isolate the mechanism to a single mode, subsequently making for more easily understood results. Consumer product constraints surrounding materials that reduce bacteria population through bactericidal mechanisms will inherently certain elicit regulatory restrictions. It is therefore important to have an understanding on materials that act as a non-lethal, almost mechanical, barrier only.

HAP-coated substrates were used as an enamel surface model to test the attachment of a mixture of oral bacteria *Actinomyces viscosus* and *Streptococcus oralis.* These bacteria are known as early colonizers of the oral biofilm formation [45,46], so significant reductions of these species are suggestive of efficacy on full healthy oral biofilms. In general, all of the polymers reduced the attachment of the bacteria onto the HAP surfaces by 17–54% relative to untreated control. However, what is immediately clear is that the six samples separated into two distinct groupings based on their relative hydrophobicity (Figure 6). Among the acrylic acid homopolymers, the linear (Lin-211) and 6-arm (6Star-129) star-shaped polymers showed a 30–32% reduction, while the 4-arm star (4Star-165) had only a 17% reduction. In contrast, all hydrophobic random copolymers gave higher percent reductions regardless of their shape. The linear copolymer (LinMA48-194), and 4-arm star-shaped copolymers (4StarMA56-215 and 4StarMA34-171) showed percent reductions of 43–54%, and were statistically superior to their homopolymer counterparts.

This data is suggestive of several points in regard to the material characteristics required for anti-attachment properties. First, while there may be small differences between the efficacies of various shapes within the homo vs. copolymer families, their effect is diminished by the presence of hydrophobic monomers within the random polymer chain. This is to say that bacteria are less able to attach themselves to the HAP surface when that surface is coated with a hydrophobic polymer, regardless of whether that polymer is linear or star-shaped. Second, the statistical groupings of the star-shaped hydrophobic polymers were near equivalent, with 34 and 56 mol.% of methyl acrylate providing similar effects. This implies that a “hydrophobic” polymer provides better anti-attachment, but no significant increases in this effect were observed over the ranges evaluated. In the future, we will explore this facet more closely as it may be possible to draw a true correlation here. In addition, the maximum amount of adsorbed polymers *q_max_* and dissociation constant *K_d_* of the homopolymer and hydrophobic copolymer with the same shape (linear, 4-, 6-arm star-shape) are very similar (Table 1). This suggests that the enhanced effect of anti-bacterial adhesion by the hydrophobicity of copolymers is not due to the difference in their inherent binding properties (the amount of polymers adhered) to HAP surfaces, but it could be related to the physicochemical properties of polymers or polymer conformations on the surface. It has been previously reported that random, block, cross-linked amphiphilic copolymers effectively prevent protein adhesion and bacterial adhesion [47,48,49,50,51,52,53,54]. The proposed mechanism is that these polymers form phase separated nano-scale domains, which reduce protein adsorption and subsequent bacterial attachment [48]. These domains are smaller than the hydrophobic/hydrophilic domains of proteins so that it is difficult for proteins to adopt their conformations to match with the surface domains. While it is not clear at this point, we speculate that the copolymers in our study may also form such segregated microdomains by association of hydrophobic side chains, which may be a more dominant factor for bacterial attachment than polymer shapes. 

The ability for a polymer to provide anti-attachment effects to an oral surface can only happen if the material sufficiently first binds to the surface. The data above has demonstrated that the chemistries needed to bind and repel are not the same. A multi-arm star-shaped polymer had significantly better binding to HAP, but in contrast composition played no such dominant role. Hydrophobicity did not dramatically decrease *K_d_*, however its presence significantly decreased bacteria attachment. Taken together, a hydrophobic star-shaped material would be the ideal polymer system to both bind to enamel and repel bacteria in our model systems.

### 2.4. Water Contact Angle

The polymer binding isotherms combined with bacteria anti-attachment clearly indicate the tunability and functionality of these polymer systems. However, these materials would need to perform in the presence of the salivary pellicle that coats all oral surfaces in order to provide sufficient effects in-vivo. The pellicle is a complex mixture of proteins, deposited to the surface of enamel by salivary flow [52], and fundamentally examining and predicting the interactions of star-shaped polymers with a pellicle surface is a sufficient and ongoing challenge for our group. Polymers will interact with a pellicle layer in different ways, depending on the dominate chemistry. For example, a recent publication [55] showed that polyanions and polycations interacted differently with the pellicle, which included their penetration depth relative to the HAP-pellicle interface. Another described changes in pellicle thickness as well as antimicrobial functionality as a function of polymer deposition and interaction with pellicle-coated HAP surfaces [12]. This effect can be rationally extended to variations in hydrophobicity and shape. While the presence of a pellicle would add significant complexity, and is outside of the scope of our current study, we did choose to examine how these polymers affected the *macroscopic* properties of HAP through contact angle measurements after pre-treatment with artificial saliva in order to demonstrate a small facet of in-vivo activity. Mucin-based artificial saliva has been a substitute for human saliva in dental research [56], and we found that HAP discs first treated with artificial saliva produced sufficient surfaces allowing for consistent measurements. Significant changes in surface energies, exhibited by major differences in water droplet contact angle, would indicate positive interactions between polymers and mucin-coated surfaces. This experiment represents our first bridging data between fundamental studies and practical applications.

**Figure 7:**
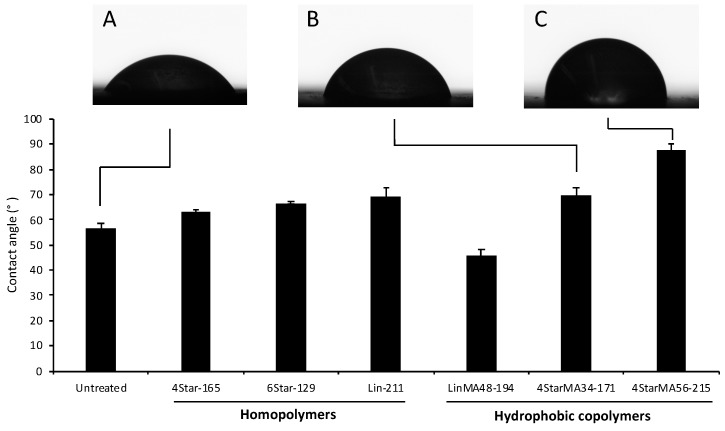
Water contact angle of polymer treated HAP surfaces pre-treated with artificial saliva. Example contact angle images of treated HAP surfaces: (**A**) untreated; (**B**) 4StarMA34-171; (**C**) 4StarMA56-215.

We generally observed that following treatment with polymer solutions, an increase in CA was observed for most samples by >7°, indicating that the polymer-treated surfaces were more hydrophobic than the untreated control (Figure 7). The magnitude of this difference also reflected the compositional changes within the polymers themselves. 4StarMA56-171, for example, had the highest contact angle of 87.6°, an effect attributed to the 56% MA concentration within the star shaped material. Example images of the droplets can be seen in Figure 7, illustrating that these materials are effective at altering the surface characteristics of HAP.

Within this series, however, the linear hydrophobic polymer LinMA48-194, exhibited a lower CA than the untreated control, even though the polymer contains 48% methyl acrylate. The hydrophobic side chains might stabilize the polymer coatings on the HAP surface by the hydrophobic interactions with HAP and/or between the polymer chains. Such polymer network anchored on the HAP surface might retain more water and therefore exhibit higher hydrophilicity, as compared to homopolymer Lin-211 which increased CA. On the other hand, the hydrophobic star-shaped polymers exhibited larger contact angles than LinMA48-194. It may be possible that linear polymer chains can adopt a conformation on the surface such that the hydrophobic side chains face down toward the HAP, and the hydrophilic (carboxylate) face up. The formation of such amphiphilic polymer conformation would be more efficient than the star-shaped polymers which have denser polymer chains, giving more constraints to conformational change. Because of the difference in the HAP pre-treatment (mucin-coated or non-coated), we cannot directly compare these results to those of binding and anti-bacterial attachment. However, the results suggest that the polymers are capable of altering the surface properties of HAP even in the presence of a protein layer.

## 3. Materials and Methods

### 3.1. Materials

2,2’-azobisisobutyronitrile (AIBN) and pentaerythritol tetrakis(3-mercaptopropionate) (PETMP) was purchased from Sigma-Aldrich Co. LLC. (St. Louis, MO, USA). Dipentaerythritol hexakis(3-mercaptopropionate) (DPEHMP) was purchased from TCI America (Montgomeryville, PA, USA). Methacryloxyethyl thiocarbamoyl rhodamine B was purchased from Polysciences (Warrington, PA, USA). Trifluoroacetic acid (TFA) and solvents were purchased from Thermo Fisher Scientific, Inc. (Waltham, MA, USA). *tert*-Butyl acrylate, methyl acrylate, and methyl mercaptopropionate (MMP) were purchased from Acros Organics (Morris County, NJ, USA). The inhibitors of these monomers were removed by passing through alumina before use. Other chemicals and solvents were used without further purification. 1H NMR was performed using a Varian MR400 (400 MHz, Agilent Scientific Instruments, Santa Clara, CA, USA) and analyzed using VNMRJ 3.2 (Agilent Scientific Instruments, Santa Clara, CA, USA) and MestReNova. Gel permeation chromatography (GPC) analysis was performed using a Waters 1515 HPLC instrument (Milford, MA, USA) using THF as an eluent, equipped with Waters Styragel (7.8 × 300 mm) HR 0.5, HR 1, and HR 4 columns in sequence and detected by a differential refractometer (RI). Sintered HAP discs (0.5 cm in diameter) were purchased from Himed, Inc. (Old Bethpage, NY, USA).

### 3.2. Synthesis of tBu PAA Homopolymers

*tert*-Butyl acrylate (t-BuA), AIBN, and chain transfer agent (CTA) (MMP, PETMP, or DPEHMP) in acetonitrile were mixed in a flask (See Table S1 for the polymerization conditions). The oxygen of the reaction mixture was removed by bubbling nitrogen gas for 10 min, and the reaction solution was stirred at 70 °C for 16 h. The reaction was cooled to room temperature. The solvent was removed by evaporation under reduced pressure. The resultant residue was dissolved in diethyl ether, and the polymer was isolated by precipitation in a methanol:water [50:50 (v/v)] mixture. The yield of purification was >90% for most cases. The polymer arm length (DP) was calculated by comparing the integrated peaks of -OCH2- group of chain transfer agent to the -CH- polymer backbone. The number average molecular weight (Mn) was calculated using the DP and molecular weights of monomers and CTAs. Gel permeation chromatography molecular mass results were determined using a calibration curve based on the standard samples of polystyrene. 1H NMR (CDCl_3_, 400 MHz) δ: 4.21–4.06 (s, 2H, –OCH_2_– of PETMP), 2.85–2.51 (brs, 4H, –SCH_2_CH_2_–), 2.37-2.07 (brs, 1H, –CH–,), 1.97–1.14 (brs, 11H, –CH_3_ and –CH_2_–). 

The tBu groups of polymers were then removed by the addition of trifluoroacetic acid (TFA) (5 mL to 1 g of polymer). After stirring for 30 min, TFA was removed by blowing with nitrogen gas in a closed container, and the gas was passed through a base (NaOH) aqueous solution to trap TFA. The residue was dissolved in methanol, and deprotected polymers were isolated by precipitating in excess diethyl ether. Subsequently, the precipitate was dissolved in distilled water and lyophilized to yield a powdery product. 1H NMR (DMSO, 400 MHz) 2.4-2.0 (brs, 1H, –CH–,), 1.8-1.2 (brs, 2H, –CH_2_–).

### 3.3. Synthesis of Random Copolymers with MA

The PAA random copolymers with methacrylate (MA) were synthesized by the same method with the tBu PAA homopolymers as described above. See Table S2 in Supporting Information for the monomer feed compositions and reaction conditions.

### 3.4. Synthesis of Rhodamine B-Labeled Polymers

The rhodamine B-labeled copolymers were synthesized using methacryloxyethyl thiocarbamoyl rhodamine B (0.1 mol.% to the total amount of monomers) by the same method as described above with the tBu PAA homopolymers. See Supporting Information for the detailed procedure, polymerization conditions, and monomer feed compositions (Tables S3–S6). The Mayo plots showed linear correlations, and the Ctr values of each thiol group of linear and 4-arm polymers are 0.91 and 0.97 (Figure S2 and Table S11).

### 3.5. Analysis of Polymerization Process

In general, *DP_arm_* of polymer prepared in the presence of thiol groups as a CTA may be presented by the Mayo equation [38]:

(2) $$\frac{1}{DP_{arm}}=\frac{1}{DP_{0}}+C_{tr}\frac{\left[SH\right]}{\left[Monomer\right]}$$

where *DP*_0_, *C_tr_*, [CTA] and [Monomer] represent the *DP* of each polymer arm in the absence of CTA, chain transfer coefficient, initial mole concentration of thiol groups, and mole concentration of monomers, respectively. According to the Mayo equation, the plot of 1/DP would be proportional to [SH]/[Monomer], and the slop presents *C_tr_*.

### 3.6. HAP Binding Assay

Fluorescence spectroscopy was used to evaluate the binding capacity of rhodamine-labeled polymers onto HAP powder. The polymer solutions in 10 mM phosphate buffer with 150 mM NaCl with different concentrations (pH = 7, adjusted by NaOH aq., 0.5 mL, 0.04, 0.08, 0.16, 0.31, 0.63, and 1.25 g/L) were mixed with HAP (30 mg/mL) in a 1.5 mL tube. The solution was gently shaken using a mechanical shaker for 2 h at room temperature and then centrifuged at 10,000 rpm for 10 min. The fluorescence emission intensities of the supernatant were measured (excitation wavelength = 553 nm, emission wavelength = 627 nm) and compared with those for samples with same concentration of polymers without HAP.

### 3.7. Anti-Bacterial Adhesion Assay

HAP coated MBECTM lids were treated by polymer solutions in MilliQ water (1 wt.%, pH 6.5 adjusted with NaOH or HCl) and allowed to shake in the incubator at 37 °C for 1 h. Following treatment, excess polymer solution was removed from the MBECTM lids by submerging in Trypticase soy broth (TSB) for 10–15 s for three cycles, replacing the TSB broth for each new cycle. The MBECTM lids were then incubated with freshly prepared overnight cultures of mixed *Actinomyces viscosus* (ATCC#43146, American Type Culture Collection, Manassas, VA, USA) and *Streptococcus oralis* (ATCC#35037, American Type Culture Collection, Manassas, VA, USA) for 3 h at 37 °C. After incubation the MBECTM lids were submerged in TSB and sonicated two times for 2 min each time in order to detach the HAP-bound bacteria into the TSB. The BacTiter-Glo Microbial Cell Viability Assay was utilized on the re-suspended TSB to determine the percent reduction in the cell viability. The percent reduction was calculated by the following equation based on the luminescent output of bacteria removed from untreated surfaces and polymer-treated surfaces: % reduction = 100 × (bacteria attached on untreated surface—bacteria attached on polymer-treated surface)/bacteria on untreated surface. 

Bartlett’s test (*p* = 0.265) suggested that any variations are not significant, and the samples have equal variances. Therefore one-way analysis of variance (ANOVA) was used to assess the treatment effect and determine the statistical differences between the various sets. A Tukey multiple comparison test was used to assess pairwise treatment differences. A *p* < 0.05 was used to indicate significant statistical differences. 

### 3.8. Contact Angle Measurements

Contact angle was performed on an Attension Theta instrument from Biolin Scientific (Stockholm, Sweden). Data was analyzed using One Attension software v 2.9. Briefly, 1.0 wt.% polymer solutions in MilliQ water were prepared, and their pH adjusted to 6.5 with concentrated NaOH or HCl. Because of the immediate absorption of solution droplets into hydroxyapatite, surface modification was required prior to treatment with polymer solutions in order to obtain stable droplets for comparison. Sintered HAP was first treated with modified artificial saliva [39] for 1 h (see Supporting Information). After this time, the discs were soaked in 2 mL of polymer solution for three hours on an orbital shaker. The discs were removed and rinsed slightly to remove excess or loosely bound material, and then dried overnight. Contact angle measurements of a 3 µL droplet on four separate HAP discs were collected and averaged to provide statistical significance.

## 4. Conclusions

In summary of the present study, we synthesized linear, 4- and 6-arm star-shaped polymers based on acrylic acid using chain transfer agents with corresponding thiol groups in order to provide insight into the types of polymers that could both bind to HAP and repel bacteria from the surface. We have found that polymer shape was more important to HAP surface binding than polymer composition (hydrophobicity). However, polymer composition played a larger role than polymer shape (linear vs. star-shape) when providing anti-bacterial protection. This information will be important for targeted properties (binding, anti-bacterial attachment, wettability, etc.,) and further design polymers for dental applications. In this study, our focus was the synthesis of star-shaped polymers and initial evaluation of their physical and biological properties in order to test new polymer platforms for dental applications. The oral environment is quite dynamic and subject to continuously changing environments due to salivary flow, food and drink intake, and the resulting fluctuating pH. Therefore, the efficiency of dental materials to provide benefits, as delivered through common oral care products, must be investigated through delivery, substantivity, and efficacy. While limited to very simple systems here, this approach is critical to build an understanding of dental materials as it can more effectively isolate and identify specific modes of action in addition to chemical or physical barriers to the effectiveness of these materials. Our future research will focus on further developing an understanding of star-shaped polymers in reference to artificial-saliva and human-saliva-coated HAP surfaces, including binding activity and bacterial anti-attachment properties.

## Figures and Tables

**Table 1:** Polymers selected for anti-bacterial adhesion assay and contact angle experiments and analogue polymers for HAP-binding assay.

Polymers Selected for Anti-Bacterial Adhesion and Contact Angle Experiments				Polymer Analogues for HAP-Binding Assay				
Polymer	DP_arm_	M_n_^a^	% Reduction	Polymer	DP_arm_	M_n_^a^	q_max_ (µmol/HAP g)	K_d_ (µM)
Lin-211	211	15,300	31.7 ± 4.2	F-Lin-189	189	13,700	1.05 ± 0.14	14.8 ± 5.5
4Star-165	165	48,000	17.6 ± 5.0	F-4Star-192	192	55,800	0.33 ± 0.06	1.4 ± 0.4
6Star-129	129	56,600	29.9 ± 2.5	F-6Star-121	121	53,100	0.38 ± 0.07	2.1 ± 0.6
LinMA48-194	194 ^b^	15,400	46.3 ± 4.3	F-LinMA51-189	189 ^b^	15,100	2.83 ± 0.11	14.0 ± 2.0
4StarMA34-171	171	53,000	43.2 ± 4.2	F-4StarMA37-185	185	57,700	0.33 ± 0.03	4.6 ± 0.8
4StarMA56-215	215	69,200	53.5 ± 5.5	F-4StarMA55-149	149	48,000	0.28 ± 0.02	1.6 ± 0.6

^a^ The Mn (the number average molecular weight) of the polymers was calculated based on the DP of protected tBu polymers and molecular weights of chain transfer agent and acrylic acrylate; ^b^ The theoretical DP calculated based on the Mayo equation using the *C_tr_* value and [SH]/[monomer].

## Supplementary Materials

The following are available online at https://www.mdpi.com/2079-4983/10/4/56/s1. Figure S1: Chain transfer process in free-radical polymerization. Figure S2: The relationships of 1/DP (A) and 1/Mn (B) with SH/monomer ratio for RhB-labeled PAA (tBu)-protected polymers. Table S1: Polymerization conditions of tBu PAA polymers. Table S2: Polymerization conditions for hydrophobic random copolymers. Table S3: Polymerization conditions for rhodamine-labeled tBu linear polymers. Table S4: Polymerization conditions for rhodamine-labeled tBu 4-arm star-shaped polymers. Table S5: Polymerization conditions for rhodamine-labeled tBu 6-arm star-shaped polymers. Table S6: Polymerization conditions of rhodamine-labeled tBu linear and 4-arm PAA/MA random copolymer. Table S7: Polymer characterization of tBu-protected polymers. Table S8: Polymer characterization of tBuA-MA random copolymers. Table S9: Polymer characterization of F-labeled polymers. Table S10: Polymer characterization and Langmuir constants. Table S11: Chain transfer constants for tBu polymers.
