# Supplementary material for: Understanding the Role of Shape and Composition of Star-Shaped Polymers and their Ability to Both Bind and Prevent Bacteria Attachment on Oral Relevant Surfaces
Source: J Funct Biomater. 2019 Dec 17;10(4):56. doi: 10.3390/jfb10040056 (PMC6963222; doi:10.3390/jfb10040056)
Supplement: Supplementary file 1 [file jfb-10-00056-s001.pdf]

Journal of *Functional Biomaterials*

Supplementary

Understanding the Role of Shape and Composition of Star-Shaped Polymers and their Ability to Both Bind and Prevent Bacteria Attachment on Oral Relevant Surfaces

Hamid Mortazavian [1], Guillaume A. Picquet [2], Jānis Lejnieks [1], Lynette A. Zaidel [2], Carl P. Myers [2,*] and Kenichi Kuroda [1,*]

[1] Department of Biologic and Materials Sciences & Prosthodontics, School of Dentistry, University of Michigan, Ann Arbor, MI 48109, USA; mortazavian.hamid@gmail.com (H.M.); jaanislejnieks@gmail.com (J.L.)
[2] Oral Care Early Research, Colgate-Palmolive Company, Piscataway, NJ 08855, USA; guillaume_picquet@colpal.com (G.A.P.); Lynette_Zaidel@colpal.com (L.A.Z.)
* Correspondence: carl_myers@colpal.com (C.P.M.); kkuroda@umich.edu (K.K.)

Received: 25 November 2019; Accepted: 13 December 2019; Published: 17 December 2019

1. Polymerization Conditions

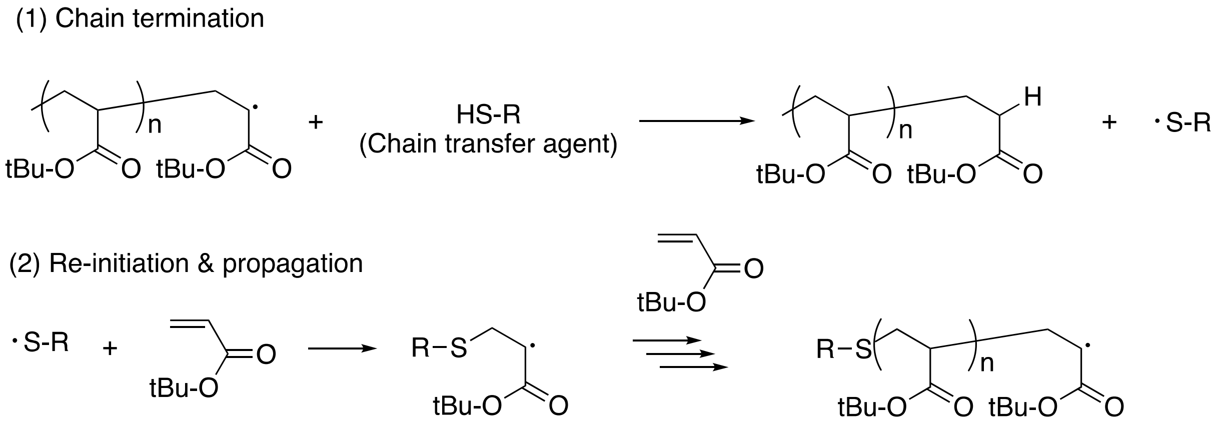

Figure S1. Chain transfer process in free-radical polymerization.

Journal of
Functional Biomaterials

MDPI

Table S1. Polymerization conditions of tBu PAA polymers.

Polymer	tBu (g)	tBu (mmol.)	CTA (g)	CTA (mmol.)	AIBN (g)/(mmol.)	AIBN (mmol.)	[CTA] /[M]	MeCN (g)	Conv. (%)	Yield (%)
Lin-27	10	78.02	0.421	0.868	0.143	0.868	0.045	20	99	-
Lin-151	10	78.02	0.060	0.126	0.021	0.126	0.006	20	99	-
Lin-323	10	78.02	0.024	0.05	0.008	0.05	0.003	20	99	-
LinPAA-238	5.0	39.01	0.032	0.039	0.006	0.039	0.007	10	-	94
LinPAA-253	20	156.04	0.130	1.56	0.256	1.56	0.007	40	-	-
Lin	5.0	39.01	0.006	0.039	0.006	0.039	0.001	10	-	90
Lin-211	5	39.01	0.024	0.201	0.027	0.039	0.0052	10	-	93
Lin-16	2.0	15.60	0.125	0.156	0.026	0.156	0.067	4	-	-
Lin-37	2.0	15.60	0.038	0.156	0.026	0.156	0.020	4	-	-
Lin-132	2.0	15.60	0.012	0.156	0.026	0.156	0.007	4	-	-
4Star-5	1.0	7.802	0.152	0.3109	0.013	0.078	0.040	2	99	100
4Star-8	1.0	7.802	0.094	0.1917	0.008	0.0479	0.025	2	99	99
4Star-11	1.0	7.802	0.068	0.1386	0.006	0.0347	0.018	2	99	99
4Star-13	1.0	7.802	0.056	0.1138	0.005	0.0285	0.015	2	99	88
4Star-16	1.0	7.802	0.046	0.0935	0.004	0.0234	0.0120	2	99	92
4Star-21	1.0	7.802	0.053	0.108	0.004	0.027	0.0138	2	-	-
4Star-4	10.0	78.02	2.425	4.962	0.204	1.241	0.064	20	99	-
4Star-39	10.0	78.02	0.312	0.639	0.026	0.16	0.008	20	99	-
4Star-130	10.0	78.02	0.123	0.252	0.010	0.063	0.003	20	99	-
4Star-18	5.0	39.01	0.257	0.525	0.064	0.39	0.013	10	-	-
4Star-165	5.0	39.01	0.024	0.05	0.064	0.39	0.001	10	-	95
4Star-196	20.0	156.04	0.098	0.2	0.256	1.56	0.001	40	-	-
6Star-3	10.0	78.020	3.535	4.515	0.185	1.128	0.058	20	99	-
6Star-21	10.0	78.02	0.407	0.52	0.021	0.13	0.007	20	99	-
6Star-24	10.0	78.02	0.159	0.203	0.008	0.051	0.003	20	99	-
6Star-12	5.0	39.01	0.418	0.534	0.006	0.039	0.014	10	-	-
6Star-129	5.0	39.01	0.042	0.054	0.006	0.039	0.001	10	-	88
6Star-5	20.0	156.04	0.170	0.2172	0.256	1.56	0.001	40	-	-

Journal of *Functional Biomaterials*

MDPI

Table S2. Polymerization conditions for hydrophobic random copolymers.

Polymer	tBuA (g)	tBuA (mmol)	MA (g)	MA (mmol)	MA % (mol.%)	CTA (g)	CTA (mmol)	AIBN (g)	AIBN (mmol)	[CTA]/[M]	MeCN (g)	Conv. (%)	Yield (%)	MA (mol.%) protected	MA (mol.%) deprotected
LinMA48-194 (theor.)	2.5	19.5	1.7	19.5	50	0.024	0.201	0.027	0.039		0.005		90	-	48
4StarMA20-171	16.0	124.8	2.69	31.2	20	0.098	0.2	0.256	1.56	0.001	40	-	-	19	20
4StarMA34-208	12.0	93.6	5.37	62.4	40	0.098	0.2	0.256	1.56	0.001	40	-	-	37	34
4StarMA56-171	8.0	62.4	8.06	93.6	60	0.098	0.2	0.256	1.56	0.001	40	-	-	56	56

Table S3. Polymerization conditions for rhodamine-labeled tBu linear polymers.

Polymer	tBuA g	t-BuA mmol	CTA g	CTA (mmol)	[CTA] /[M]	AIBN g	AIBN mmol	Rh-MA g	Rh-MA mmol	MeCN (g)	DMF (g)	Conv. (%)	Yield (%)
F-Lin-16	2.0	15.6	0.125	1.040	0.067	0.0256	0.156	0.0106	0.0156	4	2	-	-
F-Lin-50	2.0	15.6	0.037	0.312	0.020	0.0256	0.156	0.0106	0.0156	4	2	-	-
F-Lin-134	2.0	15.6	0.0125	0.104	0.007	0.0256	0.156	0.0106	0.0156	4	2	-	-
F-Lin-189	1.0	7.802	0.005	0.040	0.005	0.013	0.078	0.005	0.0078	2	1	99	91

Table S4. Polymerization conditions for rhodamine-labeled tBu 4-arm star-shaped polymers.

Polymer	tBuA g	tBuA mmol	CTA g	CTA mmol	[CTA] /[M]	AIBN g	AIBN mmol	Rh-MA g	Rh-MA mmol	MeCN (g)	DMF (g)	Conv. (%)	Yield (%)
F-4Star-19	1.0	7.802	0.051	0.105	0.013	0.013	0.078	0.005	0.0078	2	1	99	88
F-4Star-47	1.0	7.802	0.019	0.039	0.005	0.013	0.078	0.005	0.0078	2	1	-	-
F-4Star-87	1.0	7.802	0.0095	0.0195	0.003	0.013	0.078	0.005	0.0078	2	1	-	-
F-4Star-124	1.0	7.802	0.006	0.013	0.002	0.013	0.078	0.005	0.0078	2	1	-	-
F-4Star-192	1.0	7.802	0.005	0.010	0.001	0.013	0.078	0.005	0.0078	2	1	99	94

Journal of Functional Biomaterials

Table S5. Polymerization conditions for rhodamine-labeled tBu 6-arm star-shaped polymers.

Polymer	tBuA g	tBuA mmol	CTA g	CTA mmol	[CTA] /[M]	AIBN g	AIBN mmol	Rh-MA g	Rh-MA mmol	MeCN (g)	DMF (g)	Conv. (%)	Yield (%)
F-6Star-12	1.0	7.802	0.085	0.109	0.014	0.013	0.078	0.005	0.0078	2	1	99	90
F-6Star-121	1.0	7.802	0.008	0.011	0.001	0.013	0.078	0.005	0.0078	2	1	99	95

Table S6. Polymerization conditions of rhodamine-labeled tBu linear and 4-arm PAA/MA random copolymer.

Polymer	tBuA g	tBuA mmol	MA g	MA mmol	CTA g	CTA mmol	[CTA] /[M]	AIBN g	AIBN mmol	Rh-MA g	Rh-MA mmol	MeCN (g)	DMF (g)	Conv. (%)	Yield (%)
F-LinMA51-189	0.50	3.901	0.34	3.901	0.005	0.04	0.005	0.013	0.078	0.0053	0.0078	2	1	99	94
F-4StarMA18-192	0.80	6.242	0.134	1.560	0.01	0.005	0.001	0.013	0.078	0.005	0.0078	2	1	99	98
F-4StarMA37-185	0.60	4.681	0.269	3.121	0.01	0.005	0.001	0.013	0.078	0.005	0.0078	2	1	99	-
F-4StarMA55-149	0.40	3.121	0.403	4.681	0.01	0.005	0.001	0.013	0.078	0.005	0.0078	2	1	99	92

2. Characterization of Homopolymers and Random Copolymers

Table S7. Polymer characterization of tBu protected polymers.

Polymer	[CTA]/ [monomer] (×10^2)	DP_{arm} (NMR)	M_n (NMR)	M_n (GPC)	M_w (GPC)	M_w/M_n
Lin-27	4.45	27	3500	2100	4500	2.10
Lin-151	0.64	151	19400	17700	34900	1.97
Lin-323	0.26	323	41500	38400	82500	2.15
Lin	0.69	n.d.	n.d.	83900	184800	2.20
Lin-211	0.69	211	27200	22500	45800	2.03
Lin-16	0.13	16	2200	2300	4900	2.13
Lin-37	0.52	37	4900	5200	10400	2.03
Lin-132	6.67	132	17100	21600	54800	2.53
4Star-5	3.98	5	4000	3400	4000	1.19
4Star-8	2.46	8	7000	4800	6300	1.31
4Star-11	1.78	11	9200	5700	8500	1.49
4Star-13	1.46	13	10000	n.d.	n.d.	n.d.
4Star-16	1.20	16	13000	n.d.	n.d.	n.d.

4Star-21	1.38	21	11000	n.d.	n.d.	n.d.
4Star-4	6.36	4	3000	2200	2600	1.16
4Star-39	0.82	39	20000	11900	25600	2.15
4Star-130	0.32	130	67100	27200	72800	2.68
4Star-18	1.35	18	9800	7300	13100	1.8
4Star-165	0.13	165	85300	55900	111400	2.00
4Star-196	0.13	196	101000	49000	126400	2.57
6Star-3	5.79	3	3000	n.d.	n.d.	n.d.
6Star-21	0.67	21	1700	n.d.	n.d.	n.d.
6Star-24	0.26	24	19000	40800	88500	2.17
6Star-12	1.37	12	10000	7300	11800	1.62
6Star-129	0.14	129	100000	53900	115100	2.13
6Star-129	0.14	129	100000	47200	118400	2.51
6Star-238	0.69	238	184000	79800	182500	2.29
6Star-253	0.69	253	195500	80400	170200	2.12

Table 8. Polymer characterization of tBuA-MA random copolymers.

**Polymer**	**MA (mol. %)**	***DP* (NMR)**	**M_n (NMR)**	**M_n (GPC)**	**M_w (GPC)**	**M_w/M_n**
LinMA48-194	48	- [a]	-	14576	36671	2.52
4StarMA20-208	20	208	100700	61588	128174	2.08
4StarMA34-171	34	171	77500	49368	102699	2.08
4StarMA56-215	56	215	90300	30443	75959	2.49

[a] The theoretical DP calculated using the C_{tr} value and [SH]/[monomer] is 194.

3. Characterization of Rhodamine-Labeled Polymers

Table S9. Polymer characterization of F-labeled polymers.

**Polymer**	**t-Bu Polymers (Protected)**					**Acrylic Polymers**	
	**DP (NMR)**	**M_n[a] (NMR)**	**M_n (GPC)**	**M_w (GPC)**	**M_n/M_w**	**M_n[b]**	**MA (mol. %)**
Linear							

F-Lin-16	16.2	2290	2291	3269	1.43	1296	0
F-Lin-50	50.2	6550	4824	8111	1.68	3765	0
F-Lin-134	133.9	17300	15856	26704	1.68	9841	0
F-Lin-189	188.7	24500	15690	29217	1.86	13820	0
4-arm star-shaped							
F-4Star-19	18.7	10100	6636	11196	1.68	5879	0
F-4Star-47	47	24600	16613	30134	1.81	14036	0
F-4Star-87	87	46100	28024	52476	1.87	25566	0
F-4Star-124	124	64200	34900	69579	1.73	36230	0
F-4Star-192	192	99000	25440	57126	2.25	55831	0
6-arm star-shaped							
F-6Star-12	12.2	10200	6591	10554	1.6	6058	0
F-6Star-121	121.2	94000	42484	78521	1.85	53185	0
Random copolymers							
F-LinMA51-189	189 [a]	20300	12130	22957	1.89	15100	51
F-4StarMA18-131	130.7	64000	33278	66313	1.71	39482	18
F-4StarMA37-185	185.2	84000	29001	59097	2.04	57716	37
F-4StarMA55-149	149.3	63000	32336	61198	1.89	48131	55

a) Mn of protected polymers was calculated based on the DP of protected tBu polymers and molecular weights of chain transfer agent and tBu acrylate. b) Mn of deprotected acrylic polymers was calculated based on the DP of protected tBu polymers and molecular weights of chain transfer agent and acrylic acid.

4. Characterization of Polymer Binding to HA

Table S10. Polymer characterization and Langmuir constants.

Polymer	DP_{arm} [a] (NMR)	M_n[a]	MA (mol. %)	Max. Adsorption, q_{max} (mg/HAP g)	(μmol/HAP g)	K_d (mg/L)	(μM)
Linear							
F-Lin-16	16	1300	0	3.9 ± 0.5	3.04 ± 0.42	69 ±18	53.2 ±13.7
F-Lin-50	50	3800	0	9.2 ± 1.0	2.44 ± 0.27	171 ± 8	45.3 ± 2.1
F-Lin-134	134	9840	0	10.3 ± 1.4	1.05 ± 0.14	66 ± 35	6.7 ± 3.6

F-Lin-189	189	13800	0	14.5 ± 2.0	1.05 ± 0.14	205 ± 76	14.8 ±5.5
4-arm star-shaped							
F-4Star-19	19	5900	0	13.7 ± 1.6	2.34 ± 0.27	164 ± 63	27.8 ± 10.7
F-4Star-47	47	14000	0	11.1 ± 2.1	0.79 ± 0.15	30 ± 17	2.1 ± 1.2
F-4Star-87	87	26000	0	9.7 ± 0.9	0.38 ± 0.03	55 ± 37	2.2 ± 1.5
F-4Star-124	124	36200	0	10.0 ± 0.9	0.28 ± 0.02	38 ± 27	1.1 ± 0.7
F-4Star-192	192	55800	0	18.5 ± 3.1	0.33 ± 0.06	76 ± 24	1.4 ± 0.4
6-arm star-shaped							
F-6Star-12	12	6100	0	10.7 ± 2.3	1.76 ± 0.38	72 ± 30	11.9 ± 5.0
F-6Star-121	121	5320	0	20.0 ± 3.7	0.38 ± 0.07	114 ± 33	2.1 ± 0.6
Linear and 4-arm Random copolymer							
F-LinMA51-189	189 [a]	15100	51	20.0 ± 0.75	2.83 ± 0.10	99 ± 14	14.0 ± 2.0
F-4StarMA18-131	131	39500	18	16.5 ± 2.3	0.42 ± 0.06	115 ± 42	2.9 ± 1.1
F-4StarMA37-185	185	57700	37	18.8 ± 1.9	0.33 ± 0.03	263 ± 49	4.6 ± 0.8
F-4StarMA55-149	149	48100	55	13.3 ± 0.8	0.28 ± 0.02	75 ± 31	1.6 ± 0.6

(a) Mn of deprotected acrylic polymers was calculated based on the DP of protected tBu polymers and molecular weights of chain transfer agent and acrylic acid.

Journal of
Functional
Biomaterials

MDPI

5. Mayo Plots and Langmuir Constants for Rhodamine-Labeled Polymers

A.

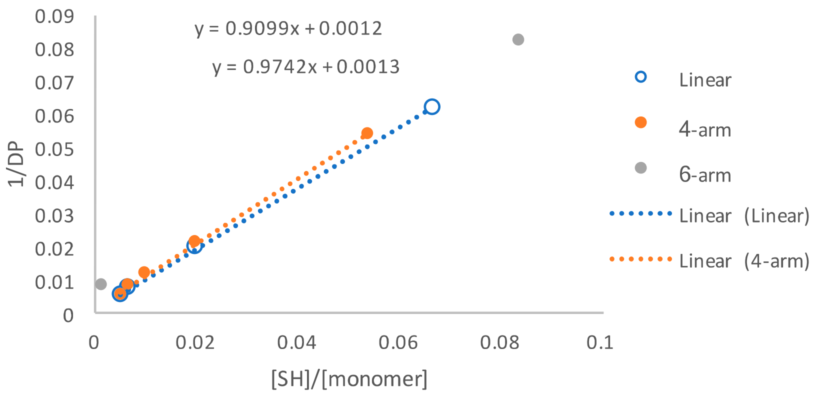

B.

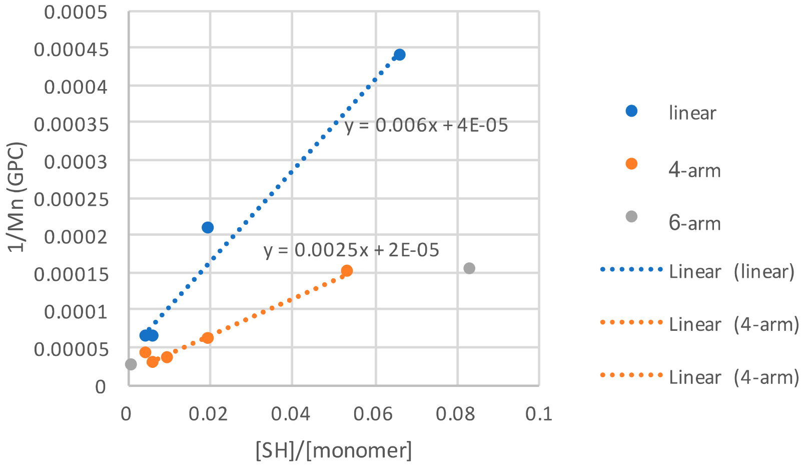

Figure S2. The relationships of 1/DP (**A**) and $1/M_n$ (**B**) with SH/monomer ratio for RhB-labeled PAA (tBu) protected polymers.

Table S11. Chain transfer constants for tBu polymers.

**Polymer**	**# of thiols** [a]	C_{tr} [b]
RhB-Lin	1	0.91
RhB-4Star	4	0.97
RhB-6Star	6	N.D.

a) The number of thiol groups of CTA. b) C_{tr} of CTA was determined by the slope of fitted line in the Mayo plots (1/DP vs. [SH]/[Monomer]).

© 2019 by the authors. Submitted for possible open access publication under the terms and conditions of the Creative Commons Attribution (CC BY) license (http://creativecommons.org/licenses/by/4.0/).
